# Optimizing Analysis Methods: Rapid and Accurate Determination of Cuaminosulfate Residues with LC-MS/MS Based on Box–Behnken Design Study

**DOI:** 10.3390/molecules29040794

**Published:** 2024-02-08

**Authors:** Mingyuan He, Yuzhu Wang, Lan Zhang, Liangang Mao, Lizhen Zhu, Yongquan Zheng, Xingang Liu, Chi Wu

**Affiliations:** 1 State Key Laboratory for Biology of Plant Disease and Insect Pests, Institute of Plant Protection, Chinese Academy of Agricultural Sciences, Beijing 100193, China; 2 Guangxi SPR Technology Co., Ltd., Nanning 530000, China; 3 State Key Laboratory of Elemento-Organic Chemistry, National Engineering Research Center of Pesticide, College of Chemistry, Nankai University, Tianjin 300071, China

**Keywords:** cuaminosulfate, dissipation, terminal residues, watermelon, soil, LC-MS/MS

## Abstract

In view of the defects in the previous detection of cuaminosulfate, which only focused on the analysis of copper ions, there is currently no analysis method available to determine the actual state of cuaminosulfate as chelated or bound. In order to investigate the dissipation and terminal residues in soil and watermelon of cuaminosulfate for food safety and environmental risk, a highly effective technique was developed to detect cuaminosulfate residues in watermelon and soil, and field experiments were conducted in China. After single-factor experiments, residual cuaminosulfate in samples was extracted by pure water, purified using a liquid–liquid approach combined with a dispersive solid-phase extraction, and detected by liquid chromatography tandem mass spectrometry (LC-MS/MS). The Box–Behnken design (BBD) study was used to find the optimal solutions for the time of liquid–liquid purification, the amount of extraction solvent, and the amounts of cleanup sorbents for the analytical method. The average recovery of the method was in the range of 80.0% to 101.1%, the average relative standard deviation (RSD) was 5.3–9.9%, and the detection limit was lower than 0.05 mg/kg. The BBD study not only improved the extraction rate of the method, but also saved time and was operated easily. The final residues of cuaminosulfate in watermelon at different sampling intervals were all lower than 0.05 mg/kg under field conditions. The cuaminosulfate in soils dissipated following exponential kinetics, with half-life values in the range of 9.39 to 12.58 days, which varied by different locations. Based on the validated method, food safety residues and soil residues can be determined rapidly and accurately.

## 1. Introduction

Watermelon is one of the most important fruit crops consumed throughout the world because it provides high nutritional value and multiple health benefits to humans [1]. For example, watermelon is rich in vitamins, which balance blood pressure and regulate heart function [2]. Approximately 3 million hectares of watermelon are grown on farmland worldwide, with an estimated global production of 101 million tons per year [3]. China is the world’s largest watermelon producer, with an annual output of 60 million tons [3]. However, watermelon is susceptible to diseases such as fusarium wilt during growth, which seriously affects fruit quality [4]. Consequently, pesticides are widely applied at different growth stages of watermelon to prevent a decline in yield [4]. Nowadays, the presence of pesticide residues in different fruits and vegetables raises serious health concerns [5]. Therefore, pesticide residues in fruits, including watermelon, should be continuously monitored to ensure consumers’ food safety and minimize the health risks [6].

**Figure 1:**
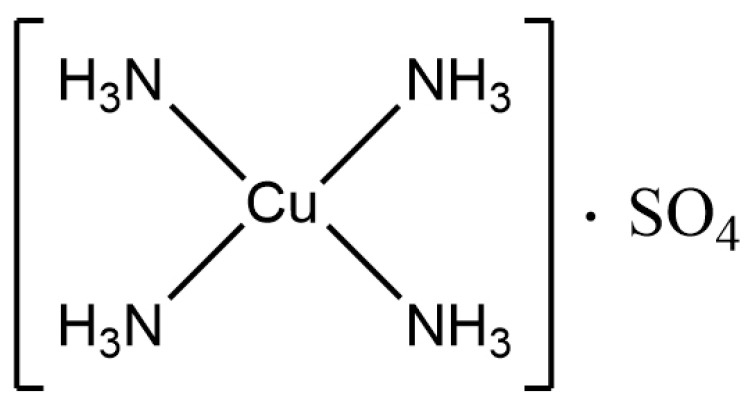
The chemical structure of cuaminosulfate.

Copper fungicide is the oldest class of pesticides [7], and is widely used in agricultural production due to its remarkable effect on prevention and control of fungal and bacterial diseases. However, current research showed that fungicides containing copper can cause adverse damage to human health [8,9,10]. Therefore, it is essential to monitor the residues of copper fungicides in crops to assess their potential threat to human health. Cuaminosulfate (Figure 1) is a broad-spectrum protective cupric fungicide used to control watermelon wilt. It can form a tight protective film on the surface of the crop, and then slowly release copper ions on the surface of the plant. The conversion of copper ions with K^+^, H^+^, and other cations on the surface of pathogen cell membrane leads to the coagulation of pathogen cell membrane proteins. At the same time, copper ions can penetrate through the cell membrane and combine with related enzymes, inhibiting the germination of pathogenic bacteria and mycelium development and affecting the activity of pathogenic bacteria [11,12,13,14].

In the past, the monitored residues of cuaminosulfate have been defined as copper ions, which are not consistent with the actual state. For example, the method reported for detecting the dissipation dynamics of cuaminosulfate in tobacco and soil focused only on copper ions, rather than chelated or bound copper compounds, and this method is easily affected by the environment and biological copper background [15]. In recent years, the guidelines of pesticide registration and management have been continually improved. Analysis of the residue of copper ions has mostly used flame atomic absorption spectrometry [15]. Residue monitoring and the toxicity assessment of copper pesticides are no longer focused on copper ions. The detection and evaluation of copper compounds in the chelated or bound state are needed to advance the detection of residues of the pesticide in crops and to establish its maximum residue limits, which are important for human health. As the most widely used analytical tool, liquid chromatography tandem mass spectrometry (LC-MS/MS) has been reported for the analysis of pesticides in the environment and food matrix [16,17,18,19].

Cuaminosulfate is insoluble in most organic solvents and easy to decompose under acidic conditions. Due to its special physical and chemical properties, pretreatments including extraction, enrichment, and clean-up of cuaminosulfate are challenging. Moreover, cuaminosulfate is widely used in fruits and vegetables, which may cause a high risk of residues after application. Therefore, there is an urgent need to establish a high sensitivity and accurate analysis method to determine the residues of cuaminosulfate complexion in various fruits such as watermelon.

Therefore, our objective in this study was to establish a rapid and effective pretreatment procedure with an analytical method to determine the presence of residues of cuaminosulfate in a chelated or bound state in watermelon and soil, while considering the efficiency and cost optimization of sulfamic acid residue analysis. Based on the QuEChERS pretreatment method, the Box–Behnken design (BBD) of the response surface method (RSM) was used to optimize the pretreatment conditions and parameters, so as to obtain better extraction and purification effects and a higher recovery rate. Moreover, the study of residue levels of cuaminosulfate in watermelon and soil under field conditions was also undertaken. Additionally, this study provides data support for the safety evaluation of pesticide residues in soil and watermelon samples, in addition to establishing a reliable residue analysis method and theoretical base.

## 2. Results and Discussion

### 2.1. Optimization of Mass Spectrometry Conditions

In the analysis of real samples at trace levels for identifying and quantifying analytes, mass spectrometry (MS) functions are used to provide structural information for identifying non-target contaminants, increase specificity for identifying target pesticides, and achieve highly sensitive trace detection [20,21,22]. The 5 mg/L individual standard solution was directly injected into the mass spectrum, and Q1 parent ion full scanning was performed for sulfamic acid under ESI positive ion mode. To obtain its protonated [M + H]^+^ as a precursor ion, different fragment voltages and different collision energies were further optimized and explored to facilitate the capture of the most abundant product ions. All of the above screening methods were used to find the most sensitive transition ions in the MRM mode as quantitative ions. Table 1 summarizes the optimal values of cuaminosulfate detection under various conditions.

### 2.2. Optimization of Chromatography

**Figure 2:**
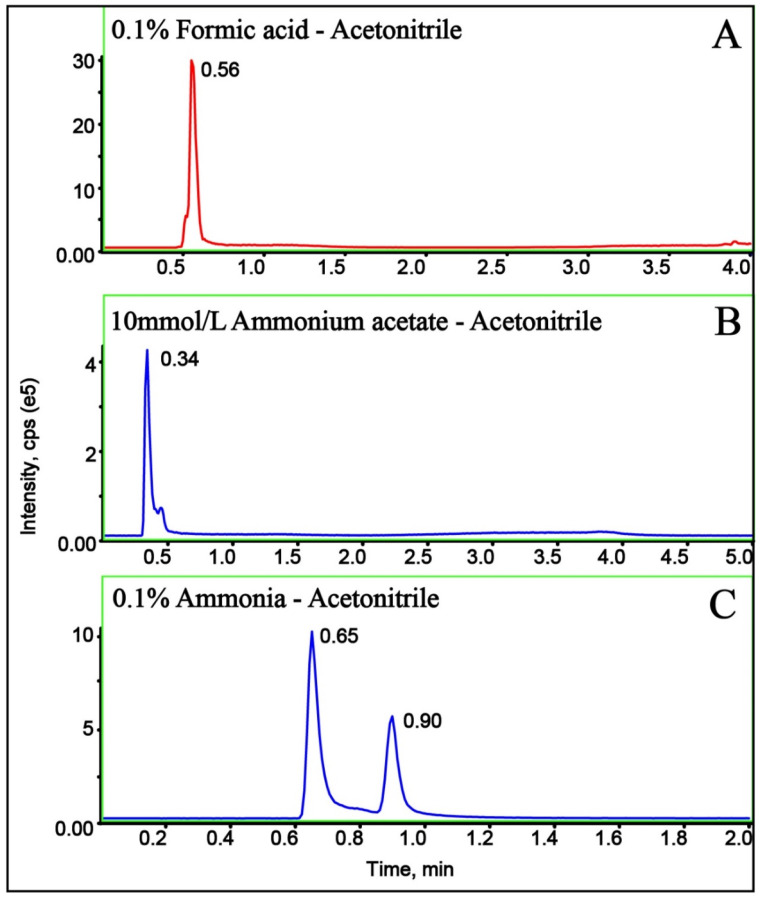
Chromatograms of cuaminosulfate with different mobile phases: (**A**) 10 mmol/L ammonium acetate-acetonitrile; (**B**) 0.1% (*v*/*v*) formic acid aqueous-acetonitrile; (**C**) 0.1% (*v*/*v*) ammonia-acetonitrile.

The mobile phase is critical in LC-MS because it affects retention time, chromatographic selectivity, ionization, detection and quantitation limits, and linear range [21,22]. The effect of pH of the mobile phase on the separation of cuaminosulfate was investigated. Certain amounts of formic acid, ammonium acetate, and ammonia water were added to the aqueous phase to adjust the pH value of the mobile phase. The chromatograms of cuaminosulfate in different mobile phases are shown in Figure 2. When 10 mmol/L ammonium acetate-acetonitrile was selected as the mobile phase, the peak shape was good, but the mass spectrum response was low (Figure 2B). It is possible that the addition of ammonium acetate inhibited the ionization of cuaminosulfate, leading to a significant decrease in MS response and a quick flowing out of the peak. When 0.1% ammonia-acetonitrile (*v*/*v*) was selected as the mobile phase, the MS response was enhanced significantly due to the obvious variations in pH. However, the chromatographic peaked shape of cuaminosulfate was not satisfactory, and was not conducive to the separation and detection of compounds (Figure 2C). When 0.1% formic acid aqueous-acetonitrile (*v*/*v*) was used, the separation and peak shape of cuaminosulfate were improved, and the ionic strength also increased significantly (Figure 2A). According to the above results, 0.1% formic acid aqueous-acetonitrile (*v*/*v*) was selected as the mobile phase for separation, which can obtain a better peak shape and stronger mass spectrum intensity.

### 2.3. Optimization of Column

**Figure 3:**
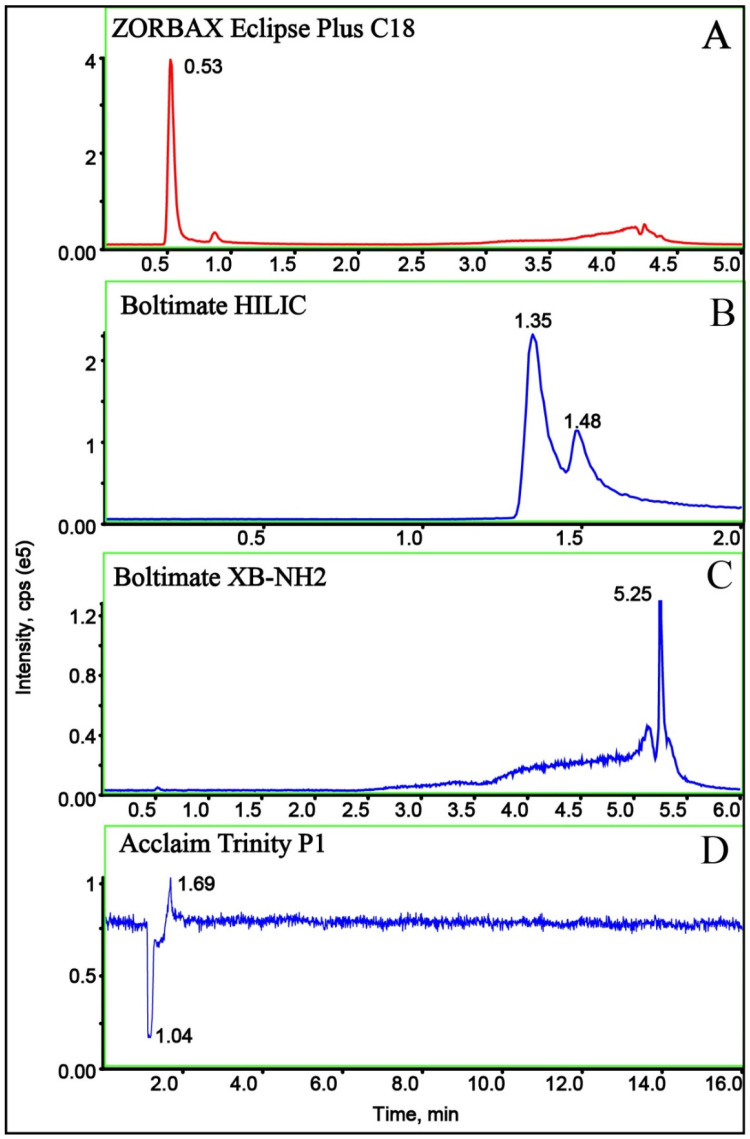
Chromatograms of chromatographic columns: (**A**) ZORBAX Eclipse Plus C18; (**B**) Boltimate HILIC; (**C**) Boltimate XB-NH2; and (**D**) Acclaim Trinity P1.

The columns selected have different separation effects. Optimizing the chromatographic method primarily involved selecting a suitable chromatographic column that could provide better retention behavior and separate target compounds from impurities [23,24]. In this study, the effects of different chromatographic columns, including Boltimate HILIC (150 mm × 21 mm, 2.7 μm), ZORBAX Eclipse Plus C18 (50 mm × 21 mm 1.8 μm), Boltimate XB-NH2 (100 mm × 30 mm, 3 μm), and Acclaim Trinity P1 (150 mm × 2.1 mm, 3 μm), on chromatographic peak shape and mass spectrum intensity were investigated. The results showed that the type of chromatographic column has a great influence on the analysis of cuaminosulfate. Boltimate XB-NH2 and Boltimate HILIC amino columns showed poor retention of cuaminosulfate and it was difficult to fully separate the impurities (Figure 3B,C). Unfortunately, the strong adsorption of the Acclaim Trinity P1 ion exchange column on cuaminosulfate led to the failure of elution from the chromatographic column, and no target chromatographic peak was observed (Figure 3D). Finally, the ZORBAX Eclipse Plus C18 (50 mm × 21 mm, 1.8 μm) chromatographic column was selected for sample separation, and a better separation effect and higher sensitivity were obtained (Figure 3A).

### 2.4. Optimization of Extraction and Cleanup Procedure

Watermelon samples are higher in moisture and sugar content than soil samples, and their matrix is more complex, making it more challenging to optimize analytical methods. Therefore, the optimization of analytical methods was focused on watermelon.

**Figure 4:**
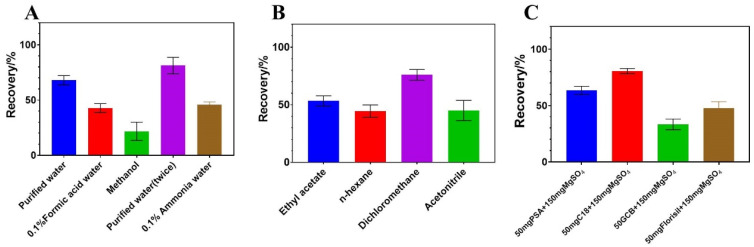
Recovery result of single-factor experiments for pretreatment procedure: (**A**) extraction solvent; (**B**) liquid–liquid purification; (**C**) cleanup sorbents.

Since QuEChERS’s high efficiency, cost effectiveness, safety, and low cost have made it an ideal pretreatment technique for analyzing residues of pesticides in a variety of matrices, it has been widely used to analyze pesticide residues in various matrices [25,26,27,28]. To ensure efficient extraction, it was essential to select a solvent based on an improved QuEChERS pretreatment. In the present experiments, pure water, methanol, 0.1% formic acid water (*v*/*v*), and 0.1% ammonia water (*v*/*v*) were studied regarding their extraction efficiencies. To evaluate the performance of the proposed method, a recovery assay was conducted, and the average recoveries were 67.9%, 81.2%, 42.7%, 45.7%, and 25.7%, respectively, for pure water, pure water (twice), 0.1% formic acid water (*v*/*v*), 0.1% ammonia water (*v*/*v*), and methanol (Figure 4A). The results showed only pure water (twice) met the requirement of recovery (70–120%) and RSDs (<20%).

The process of liquid–liquid purification can reduce the co-extraction of insoluble impurities and reduce the difficulty of subsequent purification. Therefore, we further explored the organic solvents, ethyl acetate, n-hexane, acetonitrile, and dichloromethane for liquid–liquid extraction. According to the results shown in Figure 4B, dichloromethane met the requirements of both RSD and recovery. The recoveries of other solvents were below 60%.

Purification agents are used to eliminate the organic impurities in co-extraction and achieve one-step purification. Fruits contain various sugars and carotenoids, which could dissolve in water. Multiple sorbents were selected to optimize the purification process due to their different characteristics. For example, PSA is suitable for the removal of various polar organic acids, polar pigments, and certain sugars. C18 has a strong adsorption effect on non-polar compounds [27,29,30]. Florisil is a weakly polar adsorbent, retaining less polarity as well as fewer polar compounds [31]. Four sorbents, namely, 50 mg PSA + 150 mg MgSO_4_, 50 mg C_18_ + 150 mg MgSO_4_, 50 mg Florish + 150 mg MgSO_4_, and 50 mg GCB + 150 mg MgSO_4_, were compared in this study. According to the results shown in Figure 4C, recoveries were 62.3%, 80.5%, 47.8%, and 33.3% for 50 mg PSA + 150 mg MgSO_4_, 50 mg C_18_ + 150 mg MgSO_4_, 50 mg Florish + 150 mg MgSO_4_, and 50 mg GCB + 150 mg MgSO_4_. Therefore, 50 mg C_18_ + 150 mg MgSO_4_ was used as the sorbent for the following procedure.

### 2.5. BBD Studies

As a result of single-factor experiments, different extraction solvents (pHs), liquid–liquid purifications, and cleanup procedures influenced recovery. Nevertheless, the efficiency of the pretreatment was the result of a combination of multiple factors, and it is not comprehensive to consider a single factor separately [32]. In order to obtain the optimal solution of QuEChERS conditions, we developed a three-variable, three-level BBD experiment on the volume of extraction solvent, the time of liquid–liquid purification, and the amount of cleanup sorbents (Table S2). The experiments were designed with 15 tests and 3 central points (Table S3) utilizing the software Design Expert 13.0. The results of the data analysis of BBD indicated that a quadratic regression model fits well with the data (R^2^ = 0.9766). The analysis of variance for the chosen model is shown in Table S4, which includes coefficients, F values, and *p* values. The F value for the “lack of fit” is not significant at the 95% confidence level, indicating that the model was able to accurately predict the response variables (Table S5).

**Figure 5:**
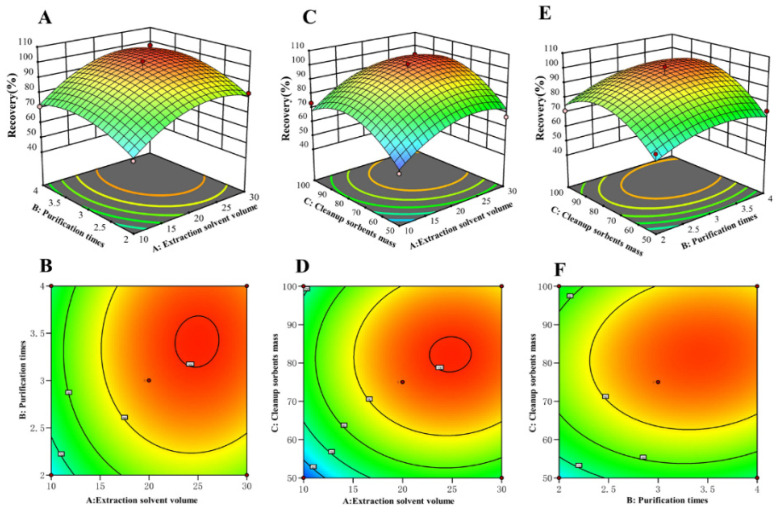
Response surfaces for Methomyl using the BBD obtained by plotting (**A**,**B**) purification time vs. extraction solvent volume, (**C**,**D**) cleanup sorbent mass vs. extraction solvent volume, and (**E**,**F**) cleanup sorbent mass vs. purification time.

Figure 5 shows a 3D response surface graph, which represents the regression equation diagram.

R = −178.48333 + 4.83417A + 42.97500B + 3.50700C + 0.115000A × B + 0.003800A × C + 0.082000B × C − 0.110667A^2^ − 7.61667B^2^ − 0.023387C^2^

where R is recovery, and A, B, and C are the volume in milliliters of extraction solvent, time of liquid–liquid purification, and milligrams of cleanup sorbents, respectively.

From 2D response surface graphs, the interaction between two factors can be found by the shape of the contour map. The shape in Figure 5D is a circle, which indicates the interaction between the volume of extraction solvent and the amount of cleanup sorbents was not significant. Alternatively, the shapes in Figure 5B,F are ovals, demonstrating that the interactions between liquid–liquid purification and amount of cleanup sorbents, volume of extraction solvent, and time of liquid–liquid purification were significant.

Table S6 displays the constraints on independent and dependent variables. In order to obtain the highest recovery, the recovery value was set to 100. Cleanup sorbents can reduce matrix effects when a sufficient quantity of sorbents is used, and the goal of factor C was set to “maximize”. Since it also impacted the response value the most (lowest *p* value), the relevance of factor C was set at 5. Meanwhile, for sake of conserving material and solvent, factors A (volume of extraction solvent) and B (time of liquid–liquid purification) were set to “minimize”. The optimum solution of BBD is displayed in Table S7, and shows that the ideal QuEChERS method for cuaminosulfate in watermelon samples was found to be 20 mL of pure water as the extraction solvent, liquid–liquid purification repeated three times, and 80 mg C18 as the cleanup sorbent with a 10 g sample.

### 2.6. Method Validation

In this study, the linearity, sensitivity, precision, and accuracy of the analytical method were validated, and the results are shown in Table 2 and Table 3. Good linearity was obtained in the range of 0.005~0.5 mg/L for cuaminosulfate, with R^2^ > 0.999 (Table 2).

In this experiment, ME was calculated based on the slope of 0.1% ammonium hydroxide and the matrix-matched calibration curves. The results showed that ME of watermelon and soil was −93.8% and −56.6%. ME means that other components in the matrix will interfere with the quantification of target analyte when they are co-ionized [32]. When |ME| ≥ 50%, ME was strong [33,34]. Therefore, the influence of the substrate on the results cannot be ignored, and the calibration should be performed for cuaminosulfate according to the external matrix-matched standards to eliminate the ME and obtain more accurate quantification in all samples.

The precision and accuracy of cuaminosulfate in watermelon and soil are shown in Table 3. The intra-day average recovery of cuaminosulfate in watermelon samples was 79.6~95.1%, and inter-day average recovery was 88.3~101.1%. The RSDr ranged from 5.3 to 9.9%, and RSD_R_ ranged from 3.5 to 9.4%, indicating that the method had high accuracy and precision. For soil samples, the intra-day and inter-day recoveries were 80.8~93.1% and 81.3~93.4%, and the RSDs were 3.4~7.8% and 4.1~7.3%, respectively, which all satisfied the requirements. The recovery results could verify the reliability of the optimal solution from the BBD study, which was a practical operation for optimizing the parameters.

After assessment of AGREEprep, a somewhat high score of 0.58 was obtained (Figure S1A). Although the preparation procedure is a green technique, some particular procedures had a few drawbacks, namely, ex situ mode and sample throughput, more waste, and the use of HPLC-MS/MS. Moreover, the blue applicability grade index (BAGI) was used as a metric tool to evaluate the practicability of method; the score was 65.0, which is higher than 60 (Figure S1B). This demonstrated that this method was considered to be practical.

### 2.7. Dissipation and Terminal Residue of Cuaminosulfate under Field Conditions

The residues of cuaminosulfate in whole watermelon at different sampling points were determined after the last application under field conditions. The average residual values of the original concentration of cuaminosulfate in watermelon were all below 0.05 mg/kg, so the dissipation kinetic fitting could not be performed. The results of final residue tests at 10 sites in 2019 showed that the median residual value (STMR) and maximum residual value (HR) of cuaminosulfate in watermelon were 0.05 mg/kg at 20, 30, and 40 days after the last application. At different harvesting intervals, the residual concentrations of cuaminosulfate in watermelon were all <0.05 mg/kg. It was also proved that there was a low risk of cuaminosulfate residues when watermelon was treated with root irrigation at the recommended dose.

To better understand the residues of cuaminosulfate in soil, the dissipation studies were undertaken in the provinces of Anhui, Guangxi, Shanxi, and Fujian in China. The initial residues of cuaminosulfate in soils ranged from 4.0 to 10.2 mg/kg, as shown in Table 4. The dissipation kinetics of cuaminosulfate in soils fitted well with the first-order kinetic equation in Fujian, Guangxi, and Anhui (R^2^ = 0.7615~0.8856). In Fujian, cuaminosulfate degraded from 9.1 to 7.4 mg/kg with a degradation rate of 77.3% in 7 days, and finally decreased to 91.6% at the end of the observation points at 28 days. In Guangxi and Guangdong, the cuaminosulfate degraded to approximately 50% at 7 days after the last application, and finally degraded to 85% and 73.7%. According to the first-order kinetic equation, the DT_50_ value of cuaminosulfate was 9.4, 12.3, and 12.6 days in Fujian, Guangxi, and Guangdong. It was demonstrated that the degradation rate was different among different locations. In Shanxi, the residues of cuaminosulfate were degraded to approximately 50% of the initial point, and then residues did not show significant differences among the following sampling points. The degradation of pesticides in the matrix may be closely related to different climatic conditions and properties of soil [35,36]. The cuaminosulfate degraded faster in Fujian compared with in other soils, which may relate to pH.

## 3. Materials and Methods

### 3.1. Reagents and Chemicals

Standard cuaminosulfate (purity 85.1%) and 15% cuaminosulfate water were obtained from Shandong United Pesticide Industry Co., Ltd. (Jinan, China) and Tianjin Huayu Pesticide Co., Ltd. (Tianjin, China) Acetonitrile (LC-MS grade) was bought from Thermo Fisher SCIENTIFIC (Shanghai, China). LC-grade formic acid was supplied by Tianjin Kermel Chemical Reagent Co., Ltd. (Tianjin, China). Methanol and n-hexane were purchased from Autogenic Bioengineering (Shanghai) Co., Ltd. (Shanghai, China) Ethyl acetate was obtained from Beijing Jinruilin Technology Development Co., Ltd. (Beijing, China) Analytical ammonium hydroxide (NH_3_·H_2_O) and dichloromethane were purchased from Xilong Scientific Co., Ltd. (Guangzhou, China) and Tianjin Fuyu Fine Chemical Co., Ltd. (Tianjin, China), respectively. Ultra-pure grade water was obtained from Hangzhou Wahaha Group Co., Ltd. (Hangzhou, China). Primary secondary amine (PSA) and octadecylsilane (C18) were obtained from Welch Materials, Inc. (Shanghai, China).

### 3.2. Standard Solution Preparation

The stock solution of cuaminosulfate (1000 mg/L) was prepared in 0.1% (*v*/*v*) ammonium hydroxide solution. The stock solution was stored in a refrigerator at 4 °C in the dark and remained stable for up to 3 months. Standard working solutions at 0.01, 0.05, 0.1, 0.2, 0.4, and 0.5 mg/L were prepared by taking appropriate aliquots of the stock solution and diluting them serially to volume in 0.1% (*v*/*v*) ammonium hydroxide solution. Correspondingly, blank sample extracts were used as solvents to prepare matrix-matched standard solutions with concentrations of 0.01, 0.05, 0.1, 0.2, 0.4, and 0.5 mg/L, respectively.

### 3.3. Chromatographic and Mass Spectrometric Conditions

Chromatographic analysis was conducted using a Waters Acquity UPLC system (Waters, Milford, MA, USA) coupled to an API 4000 triple quadrupole mass spectrometer (Sciex, Foster City, CA, USA). The separation was achieved using a ZORBAX, Eclipse Plus C18 column (2.1 mm × 50 mm, with particle size of 1.8 μm) (Agilent Technologies, Shanghai, China) at a flow rate of 0.2 mL/min. A 2 μL volume of target solution was injected into the LC–MS/MS system, and the total analysis time was 6.0 min. The mobile phase consisted of 0.1% (*v*/*v*) formic acid in water (solvent A) and acetonitrile (solvent B). Elution conditions were as follows: 0 to 1.0 min, 10% solvent B; 1.0 to 2.0 min, a linear gradient change from 10% to 90% B; 2.0 to 4.0 min, 90% B; 4.0 to 4.1 min, a linear gradient change from 90% to 10% B; and 4.1 to 6.0 min, the mobile phase returned to initial conditions. The column temperature was maintained at 35 °C.

MS detection was operated in positive ion mode. Multiple Reaction Monitoring (MRM) mode was performed on the protonated molecule for cuaminosulfate using the following optimized parameters: ion spray voltage (v): 5500; source temperature (°C): 500; curtain gas pressure (psi), 30; nebulizer gas pressure (psi), 55; auxiliary gas pressure (psi): 55; desolvation temperature (°C): 500; sheath gas pressure (psi): 9. MRM transitions and other acquisition parameters for cuaminosulfate are given in Table 1.

### 3.4. Sample Preparation

Extraction process: A quantity of 10.0 g of watermelon or soil was weighed after being ground and homogenized, then transferred to a 50 mL plastic centrifuge tube with 20 mL ultrapure water. Using a vortex mixer, the tube was vigorously shaken for 2 min. Afterward, the mixture was centrifuged at 5000 rpm for 3 min; then, the supernatant was placed in a graduated tube. The above extraction process was repeated once and the supernatant was mixed. Finally, the supernatant was diluted with ultrapure water to a final volume of 50 mL.

Liquid–liquid purification: A quantity of 4 mL of the extraction sample was transferred to a centrifuge tube, and 4 mL of dichloromethane was added. The mixture was vortexed for 2 min and centrifuged for 5 min at 4000 rpm. The above process was repeated twice and the inorganic layer was mixed.

Purified process: A quantity of 2 mL aqueous phase from the above process was transferred to a centrifuge tube containing 100 mg C18 and 20 mg PSA. The mixture was vortexed for 2 min and centrifuged for 3 min at 6000 rpm. The solution was filtered through a 0.22 μm water membrane prior to analysis.

### 3.5. Field Trial Design and Sample Collection

**Figure 6:**
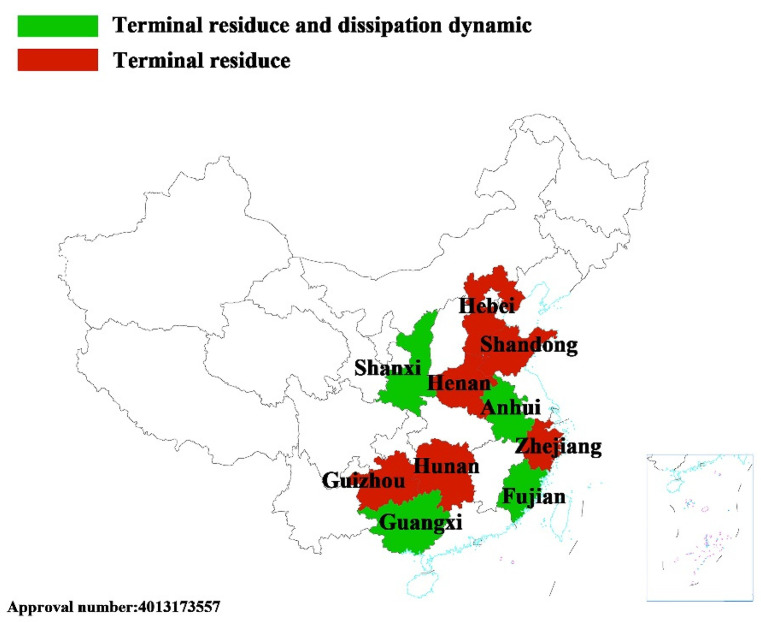
Terminal residues and dissipation dynamics of cuaminosulfate in watermelon and soil.

A field trial study was conducted at 10 sites representative of China in 2019 according to Pesticide Residue Testing Guidelines (NY/T788–2018) issued by Ministry of Agriculture and Rural Affairs of the People’s Republic of China. The 10 sites were located in Shandong, Hunan, Guangxi, Hebei, Guizhou, Shanxi, Henan, Zhejiang, Anhui, and Fujian, as shown in Figure 6. One treatment plot (50 m^2^) and one control plot (50 m^2^) were designed for each place. A conservation area of 20 m^2^ was set to separate each plot in the same field. There had never been a field trial with cuaminosulfate conducted previously. The background information is shown in Table S1.

Each plot was applied with 0.25 g active indigent/plant three times through root irrigation. Samples of watermelon and soil were collected randomly at 0, 3, 10, 20, 30, and 40 days after the last application. More than 12 disease-free and mature watermelon fruits with a total weight of at least 2 kg and soil around the collected plants were collected at each treated plot. The samples were transported to the laboratory immediately after collection. After being chopped and thoroughly mixed, samples were kept in a deep freeze (−20 °C) before analysis.

### 3.6. Method Validation

The analytical method was validated by evaluating the linearity, limit of quantification (LOQ), accuracy, precision, selectivity, and matrix effect. The specificity of the method was assessed by extracting and analyzing the blank samples of watermelon and soil.

The linearity was assessed by the correlation coefficient (R^2^) of working standard solutions with six concentrations in the range of 0.01, 0.05, 0.1, 0.2, 0.4, and 0.5 mg/L, which were obtained from standard stock solutions diluted serially by 0.1% ammonium hydroxide solution (*v*/*v*) and different matrix solutions from blank samples. The selectivity of the method was evaluated by LOQ, which was defined as the minimum spiked concentration in matrix.

Recovery tests were conducted to verify the accuracy and precision of the method. Five replications were prepared at four different spiked concentrations. The precision of the method was evaluated by intra-day (repeatability) and inter-day (reproducibility) analysis. The intra-day precision, which was used in the analysis of the spiked samples in five replicates in one day, and was calculated by the percentage of RSD (relative standard deviation), is represented by RSD_r_. The inter-day (reproducibility) analysis was conducted with the same spiked samples (five replicates) on three different days by following all the identical experimental and instrumental conditions, and the result is denoted RSD_R_.

Meanwhile, the blue applicability grade index (BAGI) and greenness of analysis were also estimated in this study to meet sustainability requirements. The analytical greenness metric for sample preparation (AGREEprep) [37] and the blue applicability grade index (BAGI) metric tool [38] were selected as the tools for the evaluation of greenness and blueness analysis.

### 3.7. Statistical Analysis

#### 3.7.1. Matrix Effect and Recovery

The matrix effect was calculated as below [39]:

ME % = (slope_m_ − slope_s_)/slope_s_ × 100%

where slope_m_ represents the slope of the matrix-matched standard curve, and slope_s_ is the slope of the solvent standard curve.

The recovery was calculated as the ratio between the peak area of the target compound in sample extracts and the peak area of a corresponding matrix-matched calibration solution. The equation is as below [40]:

Recovery (%) = Amount of pesticide obtained/Amount of pesticide added × 100%

#### 3.7.2. Dissipation Kinetics

The dissipation kinetics of cuaminosulfate in soil samples were determined using first-order kinetics according to GB/T 31270.1-2014 [41]. The equations are as follows:

C_t_ = C_0_ × e^−kt^

T_1/2_ = ln2/k

where C_t_ is the residue (mg/kg) at time t (days), C_0_ is the initial residue (mg/kg), k represents a first-order kinetic degradation rate constant, and T_1/2_ is the half-life [42].

## 4. Conclusions

In this study, a QuEChERS method based on HPLC-MS/MS for the determination of cuaminosulfate residues in watermelon was established for the first time. The influence of a single factor, i.e., extraction solvent, liquid–liquid extraction, and cleanup sorbent, on the pretreatment process was assessed, and then a BBD study of the response surface methodology determined the efficiency of combining multiple factors to obtain an optimal solution. With the BBD study, we are able to obtain a comprehensive evaluation of the optimal parameters for the multivariable pretreatment conditions. The developed method was successfully applied to determination of cuaminosulfate in watermelon and soil samples. Good linearity and satisfactory recoveries and reproducibility were obtained. The average recoveries ranged from 80.0% to 101.1%, the RSD was 5.3–9.9%, and the detection limit was lower than 0.05 mg/kg. Linearity for target compounds with satisfactory correlation coefficients (R^2^ > 0.999) was obtained in all of the matrices. Meanwhile, after evaluating the greenness and blueness of the analytical method, the score showed the method can be recommended and is practical. The results of dissipation and final residue studies showed that the residue of cuaminosulfate in watermelon was <0.05 mg/kg. The cuaminosulfate degraded fast in watermelon. In soil, cuaminosulfate dissipated following exponential kinetics with a half-life value of 9.39 to 12.58 days, which varied by location. This method was fast and reliable and should be used for checking the presence of residues in watermelon and soils, and will also help in establishing maximum residue limits (MRLs) of cuaminosulfate in watermelon in China. In future, we will investigate the effects of soil properties on the degradation rate, such as pH and soil texture.

## Figures and Tables

**Table 1:** Experimental parameters and MS conditions of cuaminosulfate in ESI+ mode.

MW	t_R_ (min)	DP (V)	Quantification Ion Transition	CE1 (eV)	Diagnostic Ion Transition	CE2 (eV)
131.67	0.51	67/65	131.9→104.0	10	131.9→122.0	6

MW: Molecular weight; t_R_: retention time; DP: declustering potential; CE: collision energy.

**Table 2:** The calibration curves, determination coefficient (R^2^), and matrix effect of cuaminosulfate.

Matrix	Calibration Curve	R^2^	Matrix Effect (%)
0.1% Ammonium hydroxide	y = 1,699,198.0640x + 19,444.5177	0.9991	-
Watermelon	y = 105,377.7778x + 4980.2778	0.9998	−93.8
Soil	y = 736,731.1573x +5217.1344	0.9981	−56.6

**Table 3:** Recovery, relative standard deviation (RSD), and LOQ of cuaminosulfate in watermelon and soil matrix.

Sample	Spiked Level (mg/kg)	Intra-Day Recovery (%)	RSDr (%)	Inter-Day Recovery (%)	RSD_R_ (%)	LOQ (mg/kg)
Watermelon	0.05	95.1	5.3	88.3	6.3	0.05
	0.5	79.6	5.4	93.2	9.4	
	1	80.0	9.9	89.4	4.9	
	2	88.3	6.5	101.1	3.5	
Soil	0.05	93.1	6.3	92.4	7.3	0.05
	0.5	88.5	7.4	87.5	4.1	
	5	80.8	7.8	83.9	4.9	
	20	81.1	3.4	801.3	6.7	

**Table 4:** Half-life (T_1/2_) for cuaminosulfate dissipation in different provinces’ field soil conditions.

Fujian					Guangxi				Shanxi				Anhui			
Collection Points	Residue (mg/kg)			Degradation Rate	Residue (mg/kg)			Degradation Rate	Residue (mg/kg)			Degradation Rate	Residue (mg/kg)			Degradation Rate
	1	2	Mean	(%)	1	2	Mean	(%)	1	2	Mean	(%)	1	2	Mean	(%)
0	8.4	9.8	9.1	-	12.8	7.6	10.2	-	3.0	5.0	4.0	-	11.0	8.0	9.5	-
7	6.7	8.1	7.4	18.9	9.6	7.4	8.5	16.6	1.0	3.0	2.0	49.8	6.0	7.0	6.5	31.5
14	2.2	2.0	2.1	77.3	4.1	7.1	5.6	45.3	2.0	1.0	1.5	62.5	5.0	5.0	5.0	47.3
21	2.6	2.1	2.4	74.0	1.0	4.0	2.5	75.2	2.0	3.0	2.5	37.8	1.0	3.0	2.0	78.8
28	0.5	1.0	0.8	91.6	2.0	1.0	1.5	85.1	2.0	2.0	2.0	50.3	3.0	2.0	2.5	73.7
Regression Equation	Y = 9.640^−0.07386x^				Y = 10.85^−0.05616x^				-				Y = 9.581^−0.05508x^			
R^2^	0.8856				0.7615				-				0.8734			
T_1/2_(d)	9.4				12.3				-				12.6			

## Data Availability

No new data were created or analyzed in this study. Data sharing is not applicable to this article.

## Supplementary Materials

The following supporting information can be downloaded at: https://www.mdpi.com/article/10.3390/molecules29040794/s1, Table S1: The field trial background information; Table S2: Different parameter and variable factor Settings for BBD; Table S3: Observation and prediction of recovery of cuaminosulfate in watermelon by BBD in 15 cycle designs; Table S4: Analysis of variance (ANOVA) of the established model; Table S5: ANOVA for quadratic model of recovery of cuaminosulfate in watermelon; Table S6: The constraints of independent variables and dependent variable; Table S7: The solution of BBD; Table S8: The supplement information of chemical substances; Figure S1: The results of AGREEprep assessment (A) and BAGI index pictograms (B).
