# Supplementary material for: Optimizing Analysis Methods: Rapid and Accurate Determination of Cuaminosulfate Residues with LC-MS/MS Based on Box–Behnken Design Study
Source: Molecules. 2024 Feb 8;29(4):794. doi: 10.3390/molecules29040794 (PMC10892704; doi:10.3390/molecules29040794)
Supplement: Supplementary file 1 [file molecules-29-00794-s001.zip › molecules-2836895-supplementary.pdf]

Supporting Information

Optimizing Analysis Methods: Rapid and Accurate Determination of Cuaminosulfate Residues with LC-MS/MS based on Box–Behnken Design Study

Mingyuan He [2,†], Yuzhu Wang [1,3,†], Lan Zhang [1], Liangang Mao [1], Lizhen Zhu [1], Yongquan Zheng [1], Xingang Liu [1] and Chi Wu [1,*]

[1] State Key Laboratory for Biology of Plant Disease and Insect Pests, Institute of Plant Protection, Chinese Academy of Agricultural Sciences, Beijing 100193, China
[2] Guangxi SPR Technology Co., Ltd., Nanning 530000, China
[3] State Key Laboratory of Elemento-Organic Chemistry, National Engineering Research Center of Pesticide, College of Chemistry, Nankai University, Tianjin 300071, China
* Correspondence: wuchi@caas.cn; Tel./Fax: +86-10-62815938
† These authors contributed equally to this work.

Table S1. The field trial background information.

Province	Soil texture	pH	Organic matter(%)	Pesticide history
Shandong	loam	7.1	1.05	/
Hunan	loam	7.9	1.29	/
Guangxi	loam	7.9	4.18	slaked lime
Hebei	sandy loam	7	2.3	cypermethrin
Guizhou	yellow brown earth	6.24	3.1	/
Shanxi	manure pile cushion	7.41	0.12	dimethomorph
Henan	fluvo-aquic soil	7.9	1.29	metribuzin
Zhejiang	blue mud	6.3	3.4	/
Anhui	clay	7.1	1.8	Imidacloprid
Fujian	humus	4.9	2.87	/

Table S2. Different parameter and variable factor Settings for BBD.

Factor	Name	parameter		
		Low (-1)	Middle (0)	High (1)
A	Extraction solvent volume (mL)	10	20	30
B	Liquid-liquid purification time (times)	2	3	4
C	Cleanup sorbents mass (mg)	50	75	100

Table S3. Observation and prediction of recovery of cuaminosulfate in watermelon by BBD in 15 cycle designs.

Run	Extraction solvent volume (mL)	Liquid-liquid purification time (times) (g)	Cleanup sorbents mass (mg)	Observed cuaminosulfate recoveries (%)	Predicted cuaminosulfate recoveries (%)
1	20	2	50	67.3	62.90
2	10	4	75	71.1	72.35
3	30	3	50	66.9	72.55
4	10	3	50	51.1	53.70
5	20	4	50	74.1	70.25

6	20	3	75	96.5	96.83
7	30	2	75	82.9	81.65
8	30	3	100	93.1	90.50
9	20	3	75	91.9	96.83
10	20	3	75	102.1	96.83
11	20	2	100	71	74.85
12	20	4	100	86	90.40
13	10	3	100	73.5	67.85
14	30	4	75	97.2	95.40
15	10	2	75	61.4	63.20

Table S4. Analysis of variance (ANOVA) of the established model.

Term	Cofficients	F value	P value
Intercept	96.83	--	--
A	5.29	0.1275	0.7357
B	3.61	0.087	0.7799
C	16.81	0.405	0.5525
A×B	452.2	10.9	0.0215
A×C	214.2	5.16	0.0723
B×C	788.85	19.01	0.0073
A×A	5.29	0.1275	0.7357
B×B	3.61	0.087	0.7799
C×C	16.81	0.405	0.5525
Lack of fit	--	1.98	0.3524

Table S5. ANOVA for quadratic model of recovery of cuaminosulfate in watermelon.

Source	Sum of Squares	*df*	Mean Square	F-value	p-value	
Model	2945.07	9	327.23	7.88	0.0175	significant
A-Extraction solvent volume	861.13	1	861.13	20.75	0.0061	
B-Liquid-liquid purification time	262.2	1	262.2	6.32	0.0536	
C-Cleanup sorbents mass	515.2	1	515.2	12.41	0.0169	
AB	5.29	1	5.29	0.1275	0.7357	
AC	3.61	1	3.61	0.087	0.7799	
BC	16.81	1	16.81	0.405	0.5525	
A^2	452.2	1	452.2	10.9	0.0215	
B^2	214.2	1	214.2	5.16	0.0723	
C^2	788.85	1	788.85	19.01	0.0073	
Residual	207.52	5	41.5			
Lack of Fit	155.33	3	51.78	1.98	0.3524	not significant
Pure Error	52.19	2	26.09			
Cor Total	3152.59	14				

Table S6. The constraints of independent variables and dependent variable.

Name	Goal	Lower limit	Upper limit	Lower weight	Upper wight	Importance
Extraction solvent volume	Minimize	10	30	1	1	4
Liquid-liquid purification time	Minimize	2	4	1	1	4

Cleanup sorbents mass	Maximize	50	80	0.1	1	5
Recovery	Target=100	70	110	1	10	5

Table S7. The solution of BBD.

Extraction solvent volume (mL)	Liquid-liquid purification time (g)	Cleanup sorbents mass (mg)	Recovery (%)	Desirability
18.046	2.593	82.417	91.669	0.745

Table S8. The supplement information of chemical substances.

Chemical substances	CAS No.	Structure
Cuaminosulfate	10380-28-6	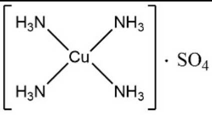
Acetonitrile	75-05-8	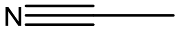
Formic Acid	64-18-6	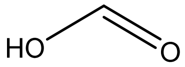
Methanol	67-56-1	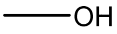
N-Hexane	110-54-3	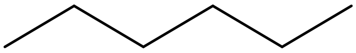
Ethyl Acetate	141-78-6	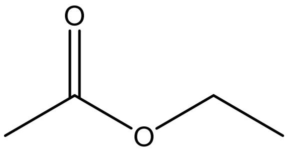
Ammonium Hydroxide	1336-21-6	NH_4^+ OH^-
Dichloromethane	75-09-2	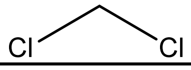

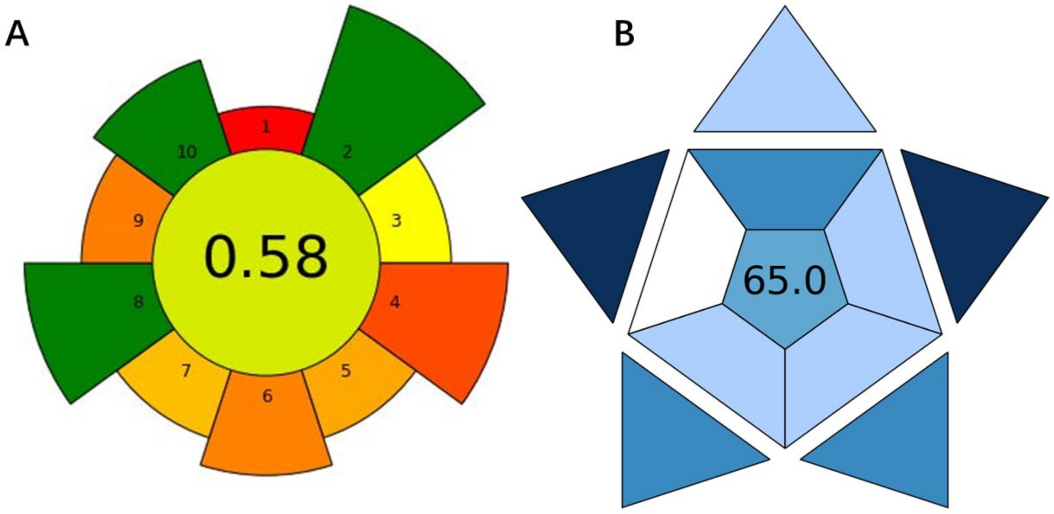

Figure S1. The results of AGREEprep assessment (A) and BAGI index pictograms (B).
